# The Possible Mechanism of Advanced Glycation End Products (AGEs) for Alzheimer’s Disease

**DOI:** 10.1371/journal.pone.0143345

**Published:** 2015-11-20

**Authors:** Shun-Yao Ko, Hshin-An Ko, Kuo-Hsiung Chu, Tzong-Ming Shieh, Tzong-Cherng Chi, Hong-I Chen, Weng-Cheng Chang, Shu-Shing Chang

**Affiliations:** 1 Graduate Institute of Medical Sciences, Collage of Health Science, Chang Jung Christian University, Tainan, Taiwan; 2 Innovate Research Center of Medicine, Chang Jung Christian University, Tainan, Taiwan; 3 Department of Food Science and Biotechnology, National Chung Hsing University, Taichung, Taiwan; 4 Department of Bioscience Technology, Collage of Health Science, Chang Jung Christian University, Tainan, Taiwan; 5 Department of Dental Hygiene, China Medical University, Taichung, Taiwan; University of S. Florida College of Medicine, UNITED STATES

## Abstract

Amyloid precursor protein (APP) has been modified by β and γ-secretase that cause amyloid deposits (plaques) in neuronal cells. Glyceraldhyde-derived AGEs has been identified as a major source of neurotoxicity in Alzheimer’s disease (AD). In a previous study, we demonstrated that glyceraldehyde-derived AGEs increase APP and Aβ via ROS. Furthermore, the combination of AGEs and Aβ has been shown to enhance neurotoxicity. In mice, APP expression is increased by tail vein injection of AGEs. This evidence suggests a correlation between AGEs and the development of AD. However, the role played by AGEs in the pathogenesis of AD remains unclear. In this report, we demonstrate that AGEs up-regulate APP processing protein (BACE and PS1) and Sirt1 expression via ROS, but do not affect the expression of downstream antioxidant genes HO-1 and NQO-1. Moreover, we found that AGEs increase GRP78 expression and enhance the cell death-related pathway p53, bcl-2/bax ratio, caspase 3. These results indicate that AGEs impair the neuroprotective effects of Sirt1 and lead to neuronal cell death via ER stress. Our findings suggest that AGEs increase ROS production, which stimulates downstream pathways related to APP processing, Aβ production, Sirt1, and GRP78, resulting in the up-regulation of cell death related pathway. This in-turn enhances neuronal cell death, which leads to the development of AD.

## Introduction

Amyloid precursor protein (APP) is modified by β and γ-secretase [1] to produce amyloid β peptide (Aβ), which accumulates in neuronal cells as amyloid deposit [2, 3]. The deposit is a pathologic characteristic of Alzheimer’s disease (AD) [4–6]. In present studies have demonstrated that BACE (beta-site APP-cleaving enzyme) and PS1 (presenilin 1) have β- and γ-secretase activity which increase Aβ [7, 8]. Ample evidence has demonstrated that oxidative stress is closely related with AD. APP processing and Aβ production are regulated by oxidative stress [9]. Moreover, Aβ leads to neuronal cell death through reactive oxygen species (ROS) [10–12].

Advanced glycation end products (AGEs) are the production of Maillard reaction between carbohydrates and proteins. In clinical setting, AGEs and receptor of AGEs (RAGE) have been identified in neurons and hippocampus [13, 14]. Recent studies have suggested that AGEs and RAGE cause neurotoxicity via oxidative stress [15–24] [25, 26]. AGEs regulate Aβ aggregation and amyloid accumulation [27, 28]. Our findings in a previous study suggested that glyceraldehyde-derived AGEs increase the expression of APP and Aβ via ROS, and that this eventually leads to cell death [29]. However, the role played by AGEs in the development of Alzheimer’s disease (AD) remains unclear.

The NAD^+^-dependent deacetylase Sirtuin 1 (Sirt1) is correlated with aging and antioxidant function. Recent studies have suggested that Sirt1 may also play a neuroprotective role [30–32], whereby it helps prevent neuronal cell death by reducing oxidative stress [33, 34]. Furthermore, the antioxidant effects of ployphenolic compounds (-)-epigallocatechin-3-gallate (EGCG) and resveratrol are activated by Sirt1 [35, 36], and a previous study involving an AD mouse model showed that Sirt1 suppresses the production of Aβ by activating α-secretase [37–39]. A number of reports have suggested that Sirt1 is able to resist the effects of AGEs [40–42]; however, the precise relationship between AGEs and Sirt1 in AD has not been previously elucidated.

AGEs have been suggested to be closely related with AD, but a complete mechanism of AGEs remains unclear. In this study, we investigate a potential link between AGEs, APP processing, antioxidant pathway and neuronal cell death pathway in AD. We demonstrate that AGEs up-regulate the expression of Sirt1 via ROS but do not affect the expression of downstream antioxidant genes HO-1 and NQO-1. We also found that AGEs increase the expression of GRP78 and enhance the cell death related pathway p53, bax/bcl-2 ratio, caspase 3. Our results suggest that AGEs impair the protective functions of Sirt1 in neuronal cells and also cause neuronal cell death via ER stress. Taken together, these findings demonstrate the important role played by AGEs in the development of AD.

## Materials and Methods

### Reagents

Bovine serum albumin (BSA), 2',7’-Dichlorodihydrofluorescein diacetate (DCFH-DA), resveratrol, and DL-Glyceraldehyde were purchased from Sigma (St. Louis, MO, USA). DMEM/F12 media, fetal bovine serum (FBS), penicillin, streptomycin, and Hanks Balanced Salt Solution (HBSS) were purchased from Invitrogen (Carlsbad, CA, USA). APP 22C11 antibody was purchased from Chemicon (Temecula, CA, USA). Sirt1, NQO-1, and GRP78 antibodies were purchased from Epitomics (CA, USA). APP, Actin antibodies, and enhanced chemiluminescence (ECL) kit were purchased from Millipore (Billerica, MA, USA); and p53, Nrf-2, Ho-1, bcl-2, bax, caspase3, and β-amyloid antibodies were purchased from Santa Cruz biotechnology (Santa Cruz, CA, USA). Nitrocellulose membranes were purchased from PALL corp. (Ann Arbor, MI, USA).

### Preparation of AGEs

AGEs were prepared by incubating BSA (pH = 7.4) in PBS with 20 mM DL-Glyceraldehyde at 37°C for 1 week. The product was dialyzed in PBS at 4°C for 2 hours, and this process was repeated five times. Using the Amicon Ultra protein concentration tube (Millipore), the solution was concentrated at 4°C before being centrifuged at 3,000 rpm for 30 min prior to storage at -80°C [29].

### Cell culture and treatment

A neuroblastoma cell line SH-SY5Y (ATCC) was grown in DMEM/F12 (1: 1) media (Invitrogen) routinely supplemented with 10% FBS, 100 units/ml of penicillin, and 100 μg/ml of streptomycin. The culture was incubated at 37°C under an atmosphere of 5% CO_2_. Prior to treatment, cells were removed from the medium and incubated serum-free for 24 hours.

### Western blot

Proteins (30 μg) were resolved using 10% SDS-PAGE gel and then transferred onto nitrocellulose membranes (PALL Corp.). The membranes were blocked using non-fat milk before being incubated with primary antibodies overnight at 4°C. Specifically, primary antibodies comprised mouse monoclonal clone 22C11 for APP (Chemicon) at a dilution of 1:1,000; rabbit monoclonal clones for Sirt1, HO-1, NQO-1, GRP78 p53, bcl-2, bax, and caspase 3 at dilutions of 1:1,000; and mouse monoclonal antibodies for Actin at a dilution of 1:10,000. Secondary antibodies included horseradish peroxidase conjugated anti-mouse, anti-rabbit, and anti-goat at a dilution of 1:1,000. Signals were detected using a Millipore enhanced chemiluminescence detection kit (Millipore). Antibody signals were quantified by normalization with the signal of Actin. Data are presented as mean values from experiments performed in triplicate.

### ROS detection

ROS levels were determined according to fluctuations in fluorescence resulting from the oxidation of DCFH-DA (Sigma) [43]. To prepare for this experiment, cells were first incubated with 20 μM DCFH-DA at 37°C for 30 min before being washed with HBSS (Invitrogen) buffer 3 times and broken with a scraper. We then transferred 100 μl of the resulting product to a 96-well plate. A fluorescence microplate reader with an excitation wavelength of 480 nm and an emission wavelength of 540 nm was used to determine the intensity of fluorescence. Data are presented as mean values from experiments performed in triplicate.

### Statistical analysis

Significant differences were analyzed using the student *t-test* and one-way ANOVA. Bars indicate the mean ± SEM obtained from experiments performed in triplicate.

## Results

### AGEs regulated APP processing via ROS

**Fig 1:**
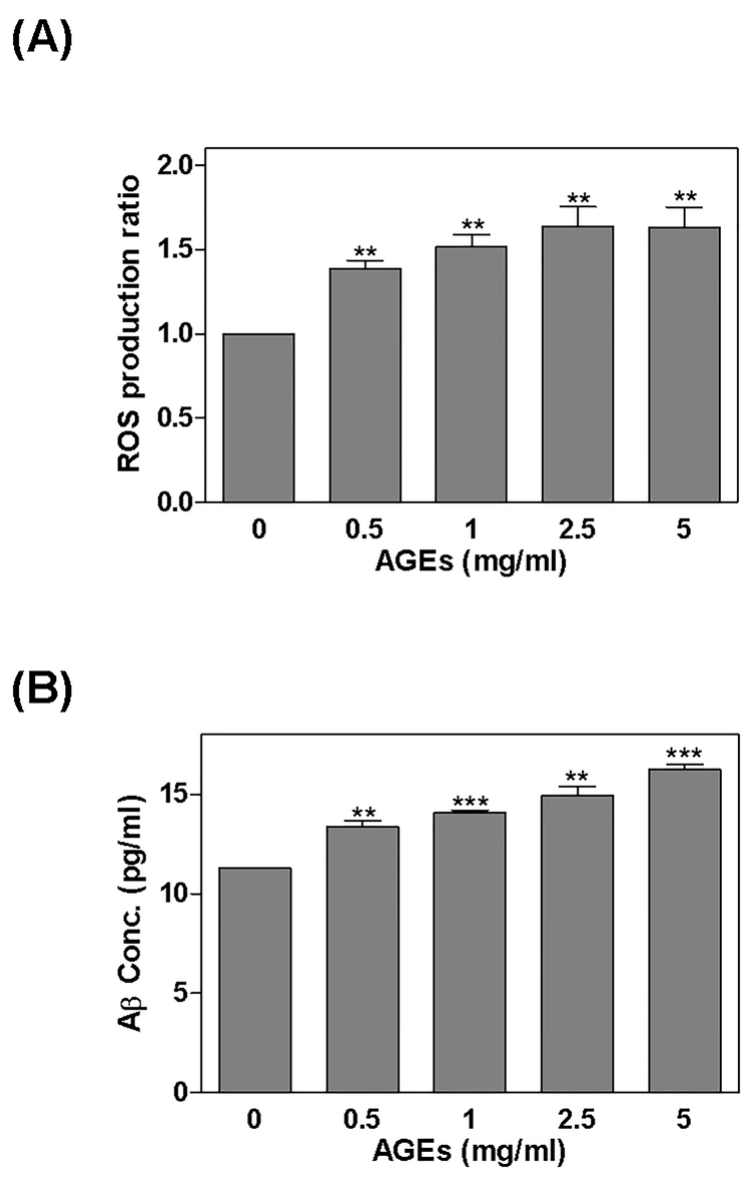
Regulation of ROS and Aβ production by AGEs. After treating cells with AGEs (0–5 mg/ml) for 24 hours: (A) ROS was detected by DCFH-DA fluorescence; and (B) Aβ_1-42_ was detected by ELISA. Note that the levels of ROS and Aβ_1-42_ increased significantly. * *P* < 0.05, ** *P* < 0.01, *** *P* < 0.001 vs. control.

**Fig 2:**
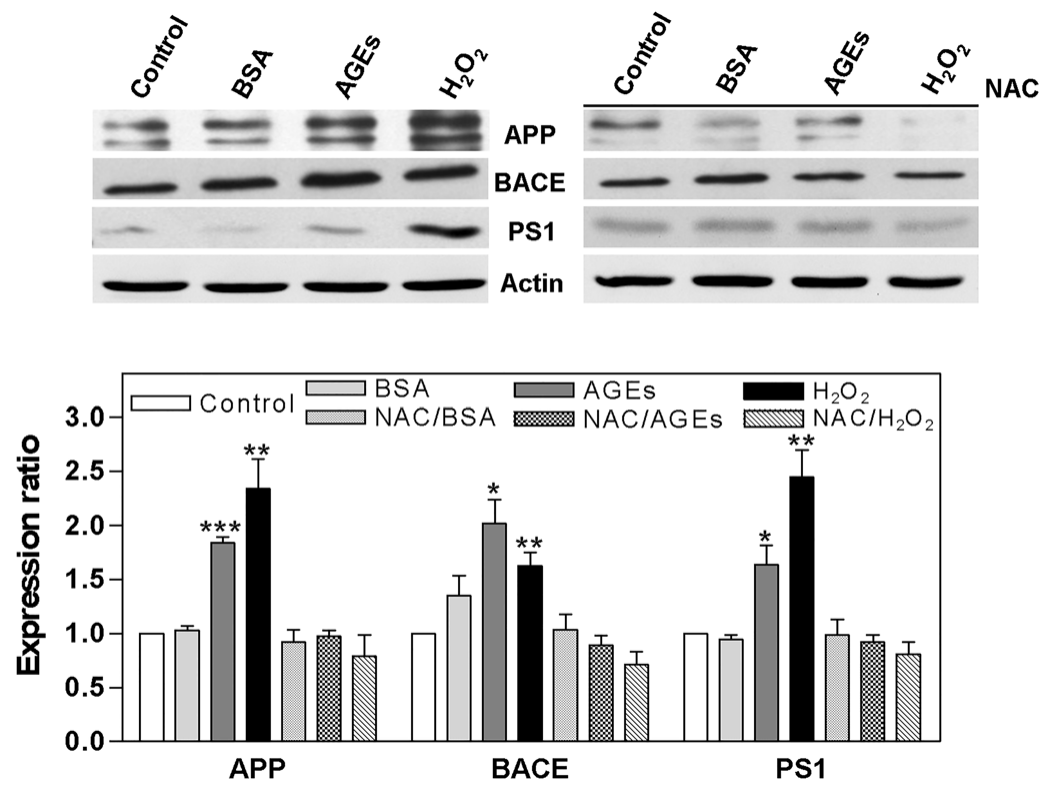
Regulation of APP processing by AGEs via ROS. Cells were treated with AGEs (0.5 mg/ml), BSA (0.5 mg/ml; as a negative control) or H_2_O_2_ (100 μM; as an ROS positive control) for 24 hours. AGEs increased the levels of APP, BACE, and PS1, and H_2_O_2_ enhanced the expression of APP, BACE, and PS1; some cells were also pretreated with ROS scavenger NAC for 2 hours before being treated with AGEs, which reduced the effects of AGEs. * *P* < 0.05, ** *P* < 0.01, *** *P* < 0.001 vs. control.

After treating cells with AGEs (0–5 mg/ml) for 24 hours, ROS was detected using DCFH-DA fluorescence (Fig 1A), and A_1-42_ was detected by ELISA (Fig 1B). ROS was found to have increased significantly to 1.39, 1.52, 1.63, and 1.63 times above normal levels. The level of A_1-42_ also increased significantly to 13.35 ± 0.32, 14.1 ± 0.1, 14.93 ± 0.48, and 16.25 ± 0.26 pg/ml (A_1-42_ was 11.27 ± 0.04 pg/ml in the control). After treating the cells with AGEs (0.5 mg/ml), BSA (0.5 mg/ml; as a negative control), or H_2_O_2_ (100 μM; as a ROS positive control) for 24 hours, we found that levels of APP (1.84 x), BACE (2.02 x), and PS1 (1.64 x) were increased by AGEs, and that H_2_O_2_ enhanced the expression of APP (2.34 x), BACE (1.62 x), and PS1 (2.45 x). Some cells were also pretreated with ROS scavenger NAC for 2 hours before being treated with AGEs, which reduced the effects of AGEs (Fig 2).

### Death related pathway was regulated by AGEs

**Fig 3:**
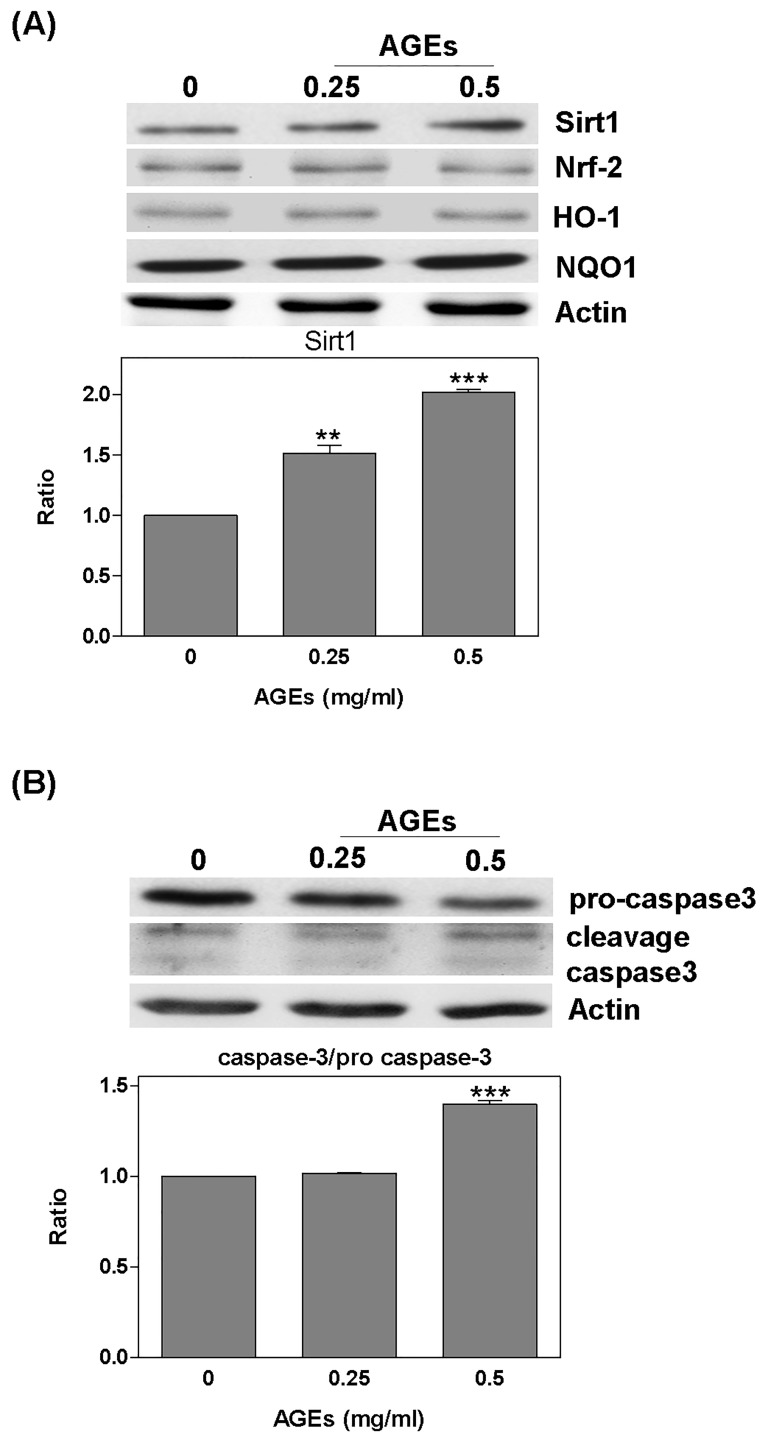
Sirt1 and caspase 3 were regulated by AGEs. Detection of Sirt1 and antioxidant protein expression after treating cells with AGEs (0–0.5 mg/ml) for 24 hours: (A) The level of Sirt1 was increased by AGEs; however, no effect was observed for Nrf-2, HO-1, or NQO-1; (B) AGEs significantly increased cleavage caspase 3. * *P* < 0.05, ** *P* < 0.01, *** *P* < 0.001 vs. control.

**Fig 4:**
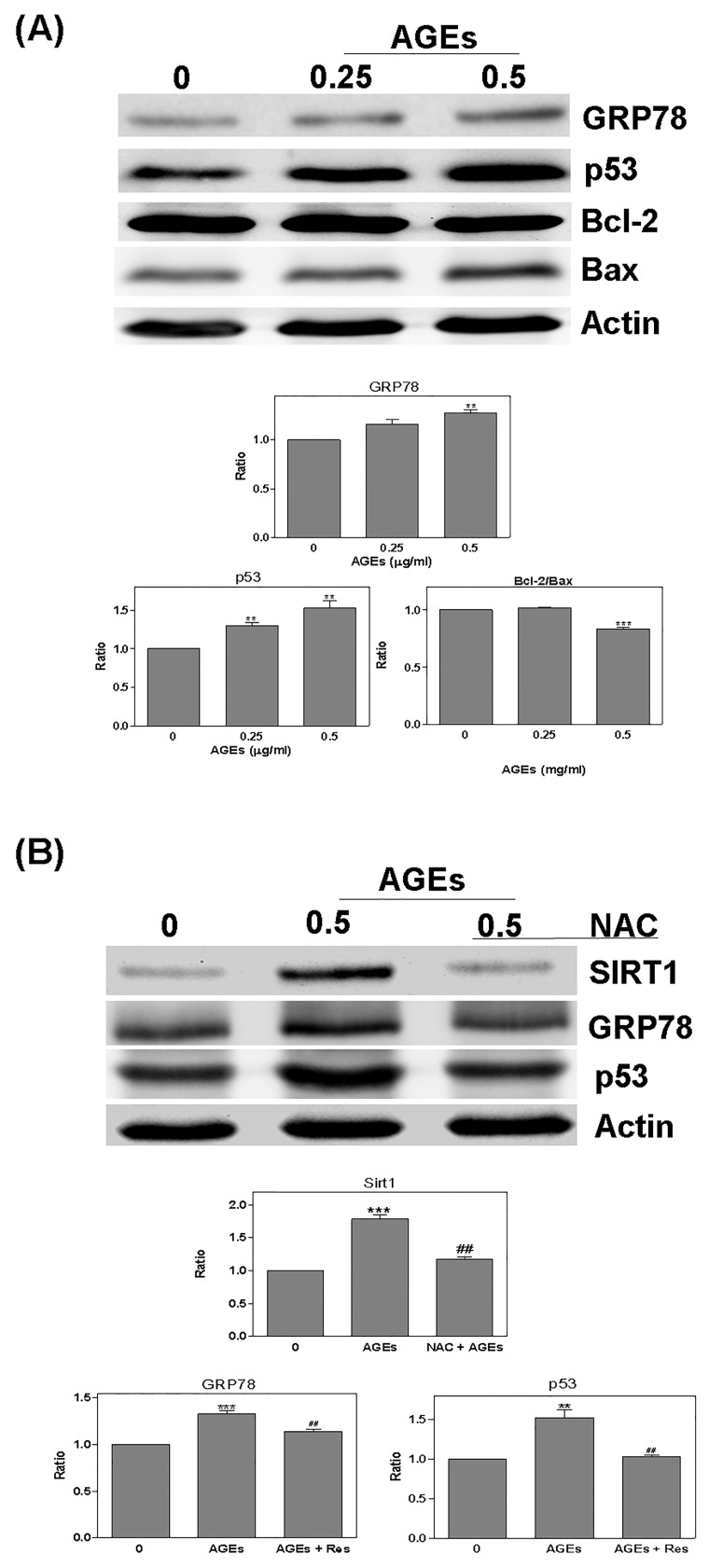
Cell death related pathway was regulated by AGEs. Detection of the death related pathway, GRP78, p53, bcl-2, and bax: (A) AGEs increased GRP78 and p53 significantly, but decreased the bcl-2/bax ratio; (B) after pretreating cells with NAC for 2 hours, the effects of AGEs were inhibited. * *P* < 0.05, ** *P* < 0.01, *** *P* < 0.001 vs. control. ^#^ *P* < 0.05, ^##^ *P* < 0.01, ^###^ *P* < 0.001 vs. AGEs.

Cells were treated with AGEs (0–0.5 mg/ml) for 24 hours prior to the detection of the expression of the antioxidant protein Sirt1. The level of Sirt1 was increased by AGEs; specifically, 0.25 mg/ml AGEs led to an increase of 1.52 x; and 0.5 mg/ml AGEs led to an increase of 2.02 x. However, the expression of Nrf-2, HO-1, or NQO-1 was no difference change (Fig 3A). AGEs were also shown to increase the fragment of caspase 3 cleavage (AGEs 0.5: 1.4 x) (Fig 3B). Furthermore, the presence of AGEs significantly increased GRP78 (AGEs 0.5: 1.28 x), p53 (AGEs 0.25: 1.3; AGEs 0.5: 1.52 x); however, it decreased the bcl-2/bax ratio (AGEs 0.5: -1.2 x) (Fig 4A). Conversely, when we pretreated the cells with NAC for 2 hours, the effects of AGEs were less pronounced (Sirt1: AGEs: 1.79 x; NAC + AGEs: 1.18 x; GRP78: AGEs: 1.33 x; NAC + AGEs: 1.14 x; p53: AGEs: 1.52 x; NAC + AGEs: 1.03 x) (Fig 4B).

### Resveratrol regulated the ROS-induced effects of AGEs

**Fig 5:**
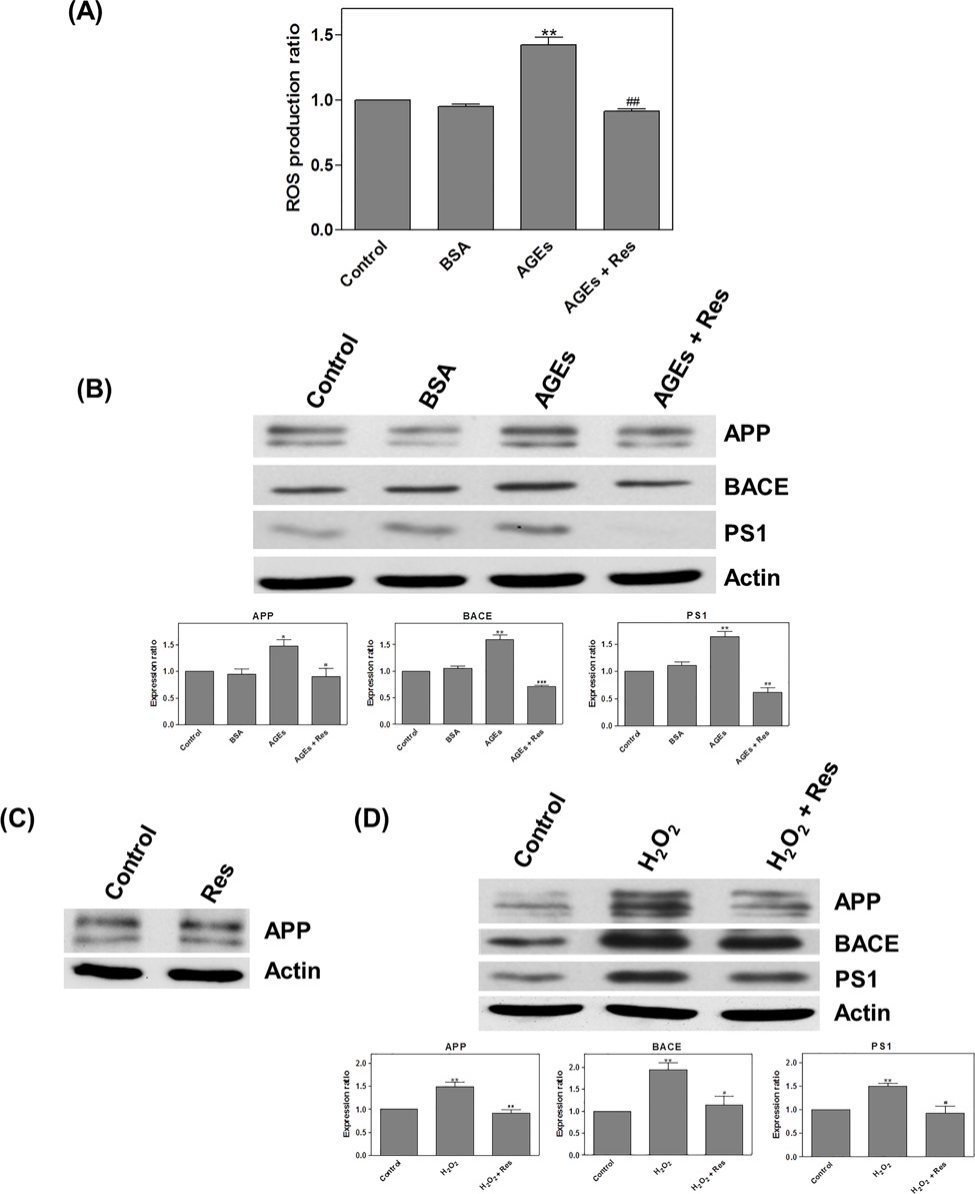
Resveratrol regulated the effects of AGEs via ROS. Detection of ROS by DCFH-DA fluorescence: (A) After treating cells with AGEs and resveratrol (Res; 20μM) for 24 hours, the level of ROS significantly increased; (B) After treating cells with AGEs the levels of APP, BACE, and PS1 were increased. (C) Treatment with resveratrol alone had no changes of APP expression. (D) H_2_O_2_ enhanced APP, BACE, and PS1 expression. (B and D) Resveratrol significantly inhibited the effects of AGEs and H_2_O_2_. * *P* < 0.05, ** *P* < 0.01, *** *P* < 0.001 vs. control. ^#^ *P* < 0.05, ^##^ *P* < 0.01, ^###^ *P* < 0.001 vs. AGEs.

Following treatment with AGEs and resveratrol (Res; 20μM) for 24 hours, we observed a significant increase in the level of ROS (AGEs: 1.42 x; AGEs + Res: 0.91 x) (Fig 5A). AGEs were also found to increase the level of APP (1.47 x), BACE (1.6 x), and PS1 (1.64 x), and in cells treated with H_2_O_2_, we observed enhanced expression of APP (1.49 x), BACE (1.94 x), and PS1 (1.5 x) (Fig 5B). Treatment with resveratrol alone had no changes of APP expression (Fig 5C). We had postulated that H_2_O_2_ (as a ROS inducer) can induce expressions of APP and its related processing proteins. And then, resveratrol significantly reduced the effects of both AGEs and H_2_O_2_ (AGEs: APP: 0.9; BACE: 0.7; PS1: 0.62 x; H_2_O_2_ APP: 0.91; BACE: 1.13; PS1: 0.93 x) (Fig 5B and 5D).

### Interaction between resveratrol and AGEs

**Fig 6:**
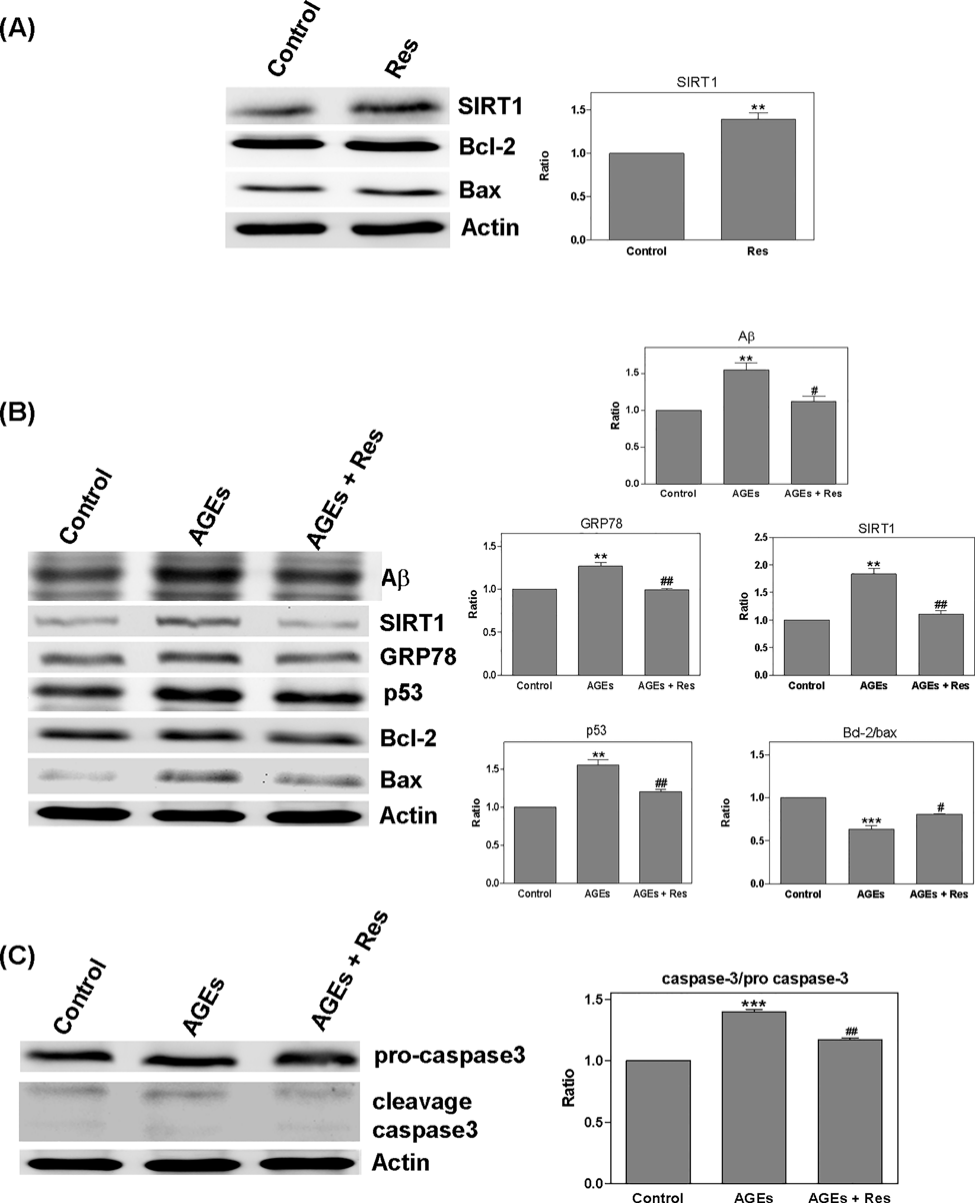
Regulation of AGEs effects by resveratrol. Detection of Sirt1, bcl-2/bax ratio after treating cells with resveratrol (Res; 10μM) for 24 hours: (A) The level of Sirt1 was increased; however, no effect was observed on the bcl-2/bax ratio; (B) Treating cells with a combination of AGEs (0.5 mg/ml) and resveratrol for 24 hours led to an increase in the bcl-2/bax ratio; (B and C) levels of Aβ, Sirt1, GRP78, p53, and cleavage caspase 3 decreased. * *P* < 0.05, ** *P* < 0.01, *** *P* < 0.001 vs. control. ^#^ *P* < 0.05, ^##^ *P* < 0.01, ^###^ *P* < 0.001 vs. AGEs.

**Fig 7:**
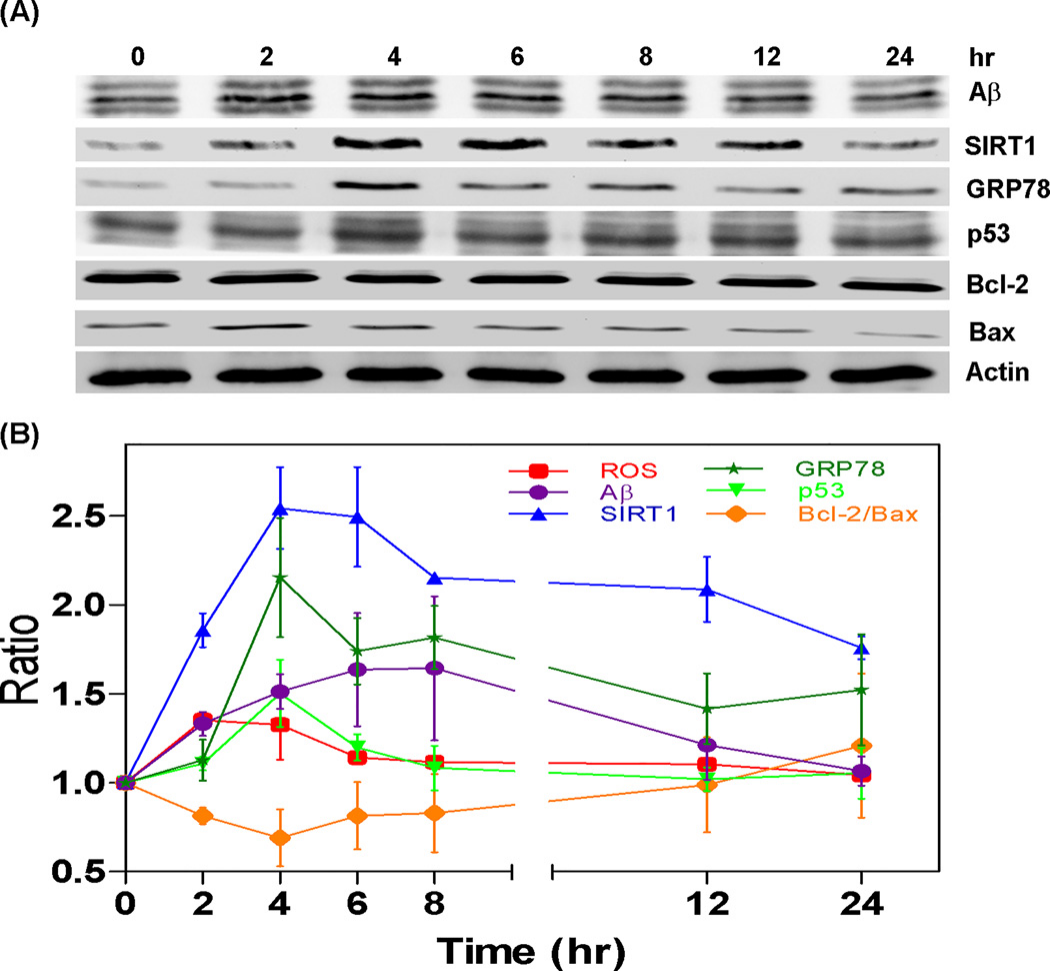
The interaction between Resveratrol and AGEs. Detection of ROS, Aβ, Sirt1, GRP78, p53, and bcl-2/bax ratio after treating cells with a combination of AGEs and resveratrol for 0–24 hours: (A and B) The level of ROS increased at 2–6 hours; Sirt1 expression increased at 2–12 hours; at 6–24 hours, the bcl-2/bax ratio was enhanced and expressions of Aβ, p53, and GRP78 were decreased.

After treating cells with resveratrol (Res; 10μM) for 24 hours, we sought to detect the quantity of Sirt1 and determine the ratio of bcl-2/bax. Resveratrol treatment increased the level of Sirt1 (1.39 x); however, it did not appear to affect the bcl-2/bax ratio (Fig 6A). Conversely, treating cells with a combination of AGEs (0.5 mg/ml) and resveratrol for 24 hours increased in the bcl-2/bax ratio (AGEs: 0.63; AGEs + Res: 0.81 x); however, this led to a reduction in A (AGEs: 1.55; AGEs + Res: 1.12 x), Sirt1 (AGEs: 1.84; AGEs + Res: 1.11 x), GRP78 (AGEs: 1.27; AGEs + Res: 0.99 x), p53 (AGEs: 1.55; AGEs + Res: 1.2 x), and cleavage caspase 3 (AGEs: 1.4; AGEs + Res: 1.17 x) (Fig 6B and 6C). Interestingly, treating cells with a combination of AGEs and resveratrol for 24 hours led to a decrease in Sirt1 expression, but the bcl-2/bax ratio was still increased. We then treated cells with a combination of AGEs and resveratrol for a specific time course (0–24 hours) and investigated the effects of the treatment on ROS, A, Sirt1, GRP78, p53, and the bcl-2/bax ratio. At 2–6 hours, the quantity of ROS was shown to increase, whereas it took 2–12 hours for the expression of Sirt1 to increase. Between 6 and 24 hours, the bcl-2/bax ratio increased whereas the expressions of Aβ, p53, and GRP78 all presented a decrease. All analyses were conducted using one-way ANOVA with results deemed statistically significant at *P* < 0.0001 (Fig 7A and 7B).

## Discussion

APP is a precursor of Aβ, which is a pathogenesis of amyloid deposit in AD. In a previous study, we demonstrated that AGEs up-regulate APP via ROS and enhance the production of Aβ [29]. Nonetheless, the mechanism underlying the relationship between AGEs and AD has yet to be elucidated. This is the first study to investigate a potential link between AGEs, APP processing and neuronal cell death pathway in AD. Our data show that AGEs increase BACE and PS1 expression, and it is suppressed by NAC pretreatment. The same discuss has been demonstrated oxidation stress inference APP processing and Aβ production [44]. This suggests that AGEs regulate APP and APP processing pathway to increase Aβ through ROS.

Previous researchers have suggested that a relationship may exist between Sirt1 and ROS in neuronal cells [33–36]. Sirt1 plays a neuroprotective function by inhibiting the effects of ROS [30–34]. Sirt1 has also been shown to decrease the production of Aβ through the activation of α-secretase [37–39, 45]. Our results reveal that AGEs increase the expression of Sirt1; however, AGEs do not appear to affect the antioxidant proteins Nrf-2, HO-1, NQO-1. Furthermore, we observed a decrease in the bcl-2/bax ratio with an increase in the expression of p53 and cleavage caspase3. We also found that AGEs increased the expression of APP, Aβ, and ROS. Our findings differ from those of previous reports, which suggested that Sirt1 does not have a neuroprotective function. For example, Yuyun et al. claimed that the high expression of Sirt1 may compromise the mass and function of mitochondria through the overproduction of ROS [46], which suggests that AGEs increase the expression of Sirt1 but also promotes cell death pathway.

Recent studies have suggested that Aβ induces stress-mediated apoptosis in the endoplasmic reticulum (ER) during AD progression [47]. In this study, we observed that AGEs up-regulated GRP78, p53, and caspase 3 and a decrease in the bcl-2/bax ratio. Lin et al. suggested that ER-stress stimulates the expression of p53 [48], and enhances the expression of GRP78, thereby inducing the cell death pathway [49] and promoting neurotoxicity [50]. Moreover, Aβ has been shown to regulate the transcription of p53 [51]. These reports confirm that AGEs cause the production of ROS and Aβ as well as the downstream ER stress-mediated apoptosis pathway. In contrast, a number of reports have suggested that Sirt1 and ER stress are antagonistic [52, 53]. Our data indicates that AGEs increase the expression of Sirt1 and GRP78, which implies that they are closely related to the neuronal cell death pathway.

Resveratrol has a direct sirtuin activation function [54], and regulate suppress ROS production [55–57]. Moreover, resveratrol has been shown to suppress the neurotoxicity of ROS and Aβ [58] via β -secretase inhibition [59]. Our results show that APP, BACE, and PS1 are increased by AGEs or H_2_O_2_, however, it is decreased by resveratrol treatment. Our data conform to resveratrol inhibit ROS, and inhibit AGEs effecting through ROS. Moreover, Our results reveal that Sirt1 and the bcl-2/bax ratio are enhanced by resveratrol. Interestingly, treating cells with a combination of AGEs and resveratrol for 24 hours led to a decrease in Sirt1 expression, but the bcl-2/bax ratio was still increased. Furthermore, when cells were treated with AGEs and resveratrol for time course of 2 to 24 hours, the level of ROS, Aβ, Sirt1, GRP78, p53, and bcl-2/bax ratio are dynamic change the same. This suggests that 2 to 12 hours, resveratrol induces the suppression of ROS and the downstream pathway by Sirt1. Our findings also confirm that AGEs and resveratrol decrease Sirt1 at 24 hours (Fig 6B). More importantly, they demonstrate that the effect of AGEs on Sirt1 and GRP78 regulation is correct.

**Fig 8:**
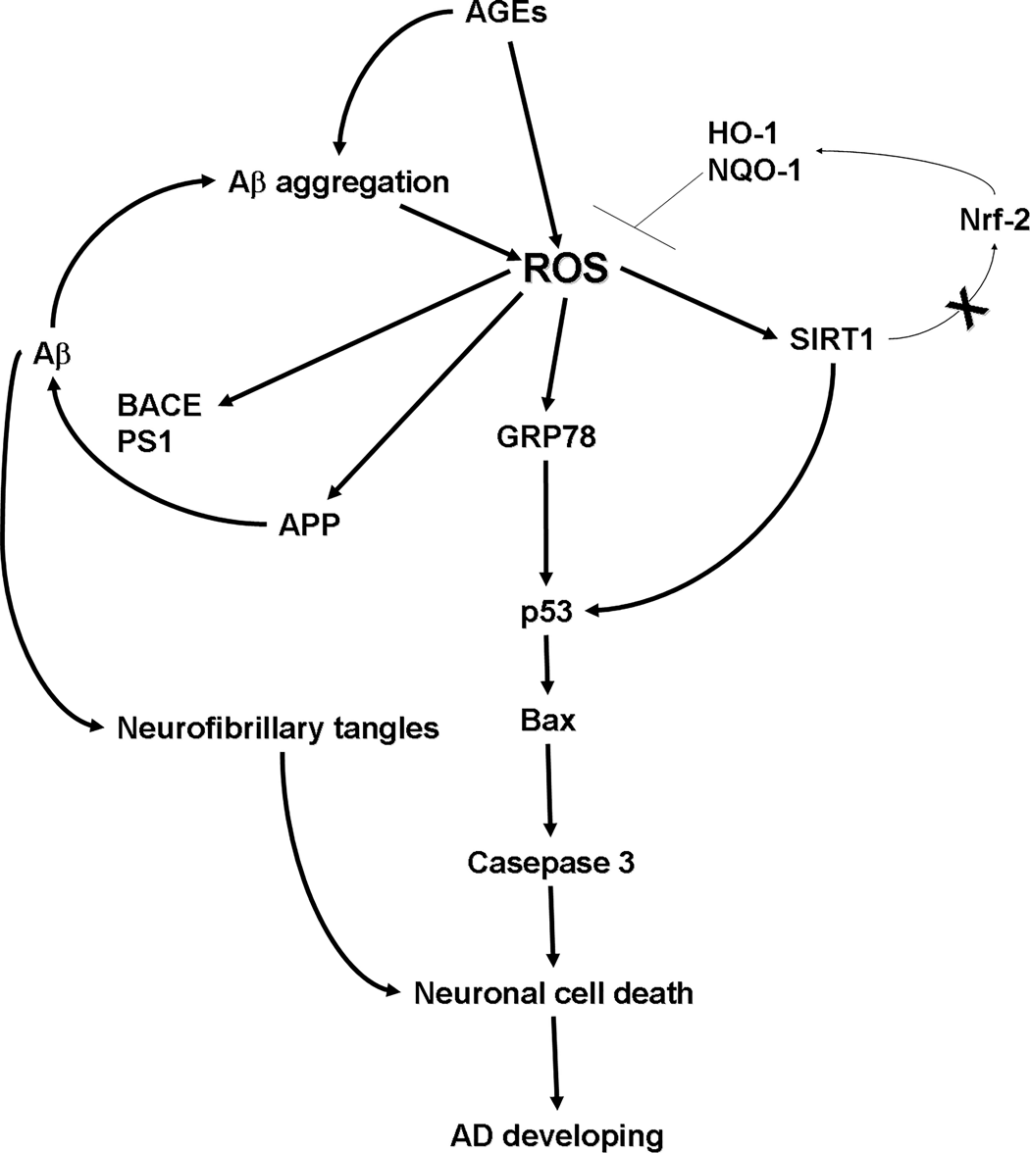
The mechanism by which AGEs promotes the development of AD. An increase in ROS production stimulated downstream pathways related to APP processing, Aβ production, Sirt1, and GRP78. This resulted in the up-regulation of the cell death related pathway and enhanced neuronal cell death, which can lead to the development of AD.

In this research, we demonstrated that AGEs increase the expression of Sirt1 and GRP78, while promoting cell death pathways. This is the first study to demonstrate that AGEs regulates the expression of Sirt1 and GRP78 via ROS. More importantly, we were able to elucidate the mechanism played by AGEs in the pathogenesis of AD through the regulation of ROS. The mechanism by which AGEs affect the development of AD is presented in Fig 8. As shown in the figure, AGEs increase the production of ROS, which stimulates the downstream pathways of Sirt1, GRP78, APP processing, and Aβ production. Fourthermore, the formation of Aβ aggregation and neurofibrillary tangles are enhanced. This in-turn ultimately results in the up-regulation of the cell death pathway, which enhances neuronal cell death, leading to the development of AD. The accumulation of AGEs is a natural process that increases slowly with aging; however, abnormal accumulation of AGEs can be induced by disease, dietary habits, and other factors. AGEs rapidly accumulate in the body, then through in the brain, and turn on the mechanism of AGEs effecting. Finally, in life span, the level of AGEs is an important key point for AD developing.
